# Assessing introgressive hybridization in roan antelope (*Hippotragus equinus*): Lessons from South Africa

**DOI:** 10.1371/journal.pone.0213961

**Published:** 2019-10-18

**Authors:** Anna M. van Wyk, Desiré L. Dalton, Antoinette Kotzé, J. Paul Grobler, Prudent S. Mokgokong, Anna S. Kropff, Bettine Jansen van Vuuren

**Affiliations:** 1 Department of Genetics, University of the Free State, Bloemfontein, South Africa; 2 National Zoological Garden, South African National Biodiversity Institute, Pretoria, South Africa; 3 Centre for Ecological Genomics and Wildlife Conservation, Department of Zoology, University of Johannesburg, Auckland Park, South Africa; National Cheng Kung University, TAIWAN

## Abstract

Biological diversity is being lost at unprecedented rates, with genetic admixture and introgression presenting major threats to biodiversity. Our ability to accurately identify introgression is critical to manage species, obtain insights into evolutionary processes, and ultimately contribute to the Aichi Targets developed under the Convention on Biological Diversity. The current study concerns roan antelope, the second largest antelope in Africa. Despite their large size, these antelope are sensitive to habitat disturbance and interspecific competition, leading to the species being listed as Least Concern but with decreasing population trends, and as extinct over parts of its range. Molecular research identified the presence of two evolutionary significant units across their sub-Saharan range, corresponding to a West African lineage and a second larger group which includes animals from East, Central and Southern Africa. Within South Africa, one of the remaining bastions with increasing population sizes, there are a number of West African roan antelope populations on private farms, and concerns are that these animals hybridize with roan that naturally occur in the southern African region. We used a suite of 27 microsatellite markers to conduct admixture analysis. Our results indicate evidence of hybridization, with our developed tests using a simulated dataset being able to accurately identify F1, F2 and non-admixed individuals at threshold values of *qi* > 0.80 and *qi* > 0.85. However, further backcrosses were not always detectable with backcrossed-Western roan individuals (46.7–60%), backcrossed-East, Central and Southern African roan individuals (28.3–45%) and double backcrossed (83.3–98.3%) being incorrectly classified as non-admixed. Our study is the first to confirm ongoing hybridization in this within this iconic African antelope, and we provide recommendations for the future conservation and management of this species.

## Introduction

Factors such as climate change, habitat fragmentation, and environmental degradation are influencing the distribution and abundance of species, often in ways that are impossible to predict [1]. Thus, a central theme in conservation biology is how best to manage for species preservation under rapidly changing and often unpredictable conditions. When faced with environmental change, species may persist by moving (or being moved) to track suitable environments. Although there is sufficient evidence to suggest that species notably alter their ranges [2], facilitation of such movement for larger vertebrate species (through the creation of habitat corridors, transfrontier parks or translocations) often place insurmountable burdens on conservation agencies that are ultimately responsible for the management of these populations. Notwithstanding, signatory countries to the Convention on Biological Diversity have an obligation to manage and protect biodiversity, as also set out more recently in the Aichi Biodiversity Targets.

Anthropogenic hybridization and introgression are major threats to species conservation (these threats are dealt with specifically under Aichi Target 13; see https://www.cbd.int/sp/targets/). The ability to accurately identify introgression is critical to the management of species [3–8], and may provide unprecedented insights into evolutionary processes. Although admixture, or even genetic rescue, may have beneficial outcomes through the introduction of new alleles into small or isolated populations, it can lead to outbreeding depression essentially disrupting locally adapted gene-complexes [9–12]. Because of the movement of animals (either natural or human-facilitated), admixture and the effects thereof become increasingly more important to understand and manage.

Roan antelope (*Hippotragus equinus*) is one of Africa's most iconic large antelope species. It has a sub-Saharan range, is a water-dependent species, and prefers savanna woodlands and grasslands. Ansell [13] conducted a study based on morphological analyses and recognised six subspecies namely *H*. *e*. *bakeri*, *H*. *e*. *charicus*, *H*. *e*. *cottoni*, *H*. *e*. *equinus*, *H*. *e*. *koba* and *H*. *e*. *langheldi*. However, subsequent genetic studies by Matthee and Robinson [14] and Alpers et al. [15] provided less support for these subspecies designation. Although the Robinson [14] study included relatively few specimens (only 13 animals were available at the time), Alpers et al. [15] analyzed 137 animals sampled from across the range (the only subspecies not included in this study was *H*. *e*. *bakeri*) for both the mtDNA control region and eight microsatellite markers. Both the mtDNA control region and microsatellite data provided strong support for a separation between the West Africa population (corresponding to the *H*. *e*. *koba* subspecies) and those from East, Central and Southern Africa (representing the *H*. *e*. *cottoni*, *H*. *e*. *equinus* and *H*. *e*. *langheldi* subspecies). Although some differentiation between East, Central and Southern African roan antelope was evident from mtDNA data, different subspecies did not form monophyletic groups, with no differentiation observed for microsatellite data. The placement of the two specimens from Cameroon (corresponding to the *H*. *e*. *charicus* subspecies) were unclear, and the small sample size precluded robust analyses. Based on these results, Alpers et al. [15] argued that two evolutionary significant units should be recognized for roan antelope in Africa, corresponding to a West African lineage and an East, Central and Southern African lineage.

Roan antelope is listed as Least Concern, but with decreasing population sizes, notably in East and Southern Africa [16]. In Southern Africa, roan antelope numbers have dramatically declined in Botswana, Namibia and Zimbabwe and these animals have disappeared from large parts of their former range including Angola and Mozambique [17]. In South Africa, roan antelope numbers in reserves and protected areas are critically low, with the majority of animals residing under private ownership on game farms. Indeed, the estimated population size of wild and naturally occurring roan antelope in protected areas in South Africa is less than 300 animals [18], yet indications are that roan antelope is thriving on private land. Current estimates suggest that at least 3,500 individuals are managed on private farms [19], with numbers increasing due to these animals being considered an economically important species by the South African wildlife industry. In the 1990s, a number of roan antelope (approximately 40) originating from Senegal, Ghana and Benin, were imported legally into South Africa under permit from West Africa and are confined to three farms in the Limpopo Province (South Africa). Subsequent to their import, and based on DNA evidence [15], an embargo was placed on the trade of West African animals in South Africa. Recent anecdotal evidence suggested that animals of West African decent was being illegally traded (based on mitochondrial haplotypes; Jansen van Vuuren, pers. comm.), thereby presenting a real and significant threat to the genetic integrity of roan antelope in South Africa, notwithstanding legislation prohibiting it. Furthermore, animals are sometimes being exported to other Southern African countries, further endangering regional gene pools. Following this, the standard practice in South Africa is that every animal that is sold on auction or moved between farms or provinces must be tested via nuclear and/or mitochondrial markers before a permit is obtained.

Our aim here is to expand on the limited and non-specific suite of microsatellite markers employed by Alpers et al. [15]. We also assessed the ability of these markers to discriminate between non-admixed animals and hybrid offspring. Our results will not only confirm whether suggestions of hybridization are true, but will also provide a valuable tool to ensure genetic integrity in the conservation of roan antelope on private farms in Southern Africa.

## Materials and methods

### Sampling

Blood, tissue or hair material was obtained from private breeders and game farm owners throughout South Africa (Table 1). Western roan reference samples were selected from the Alpers et al. [15] study and were supplemented with animals of confirmed geographic origin (all Western roan antelope samples were obtained during or before 2004). Reference samples representing the East, Central and Southern African ESU were from farms that never imported Western roan onto their property and therefore has a history of purity. A total of 32 West African roan antelope (isolated populations, with confirmed West African roan antelope mitochondrial lineage), and 98 animals representing the East, Central and Southern African ESU (populations from two farms in the Northern Cape and North West provinces, South Africa) were included. In addition, eight known hybrids and 15 putative hybrids were included in this study (Table 1), provided to us by game owners that legally had West African roan on their farms. Ethical approval was obtained from the Animal Research Ethics Committee, University of the Free State, South Africa (UFS-AED2017/0010) and the NZG Research Ethics and Scientific Committee (NZG/RES/P/17/18). Samples were stored in the NZG Biobank and access for research use of the samples was approved under a Section 20 permit from the Department of Agriculture, Forestry and Fisheries, South Africa (S20BB1917).

**Table 1:** List of roan antelope (*Hippotragus equinus*) samples.

Population / Province	Sample size	Classification
Western roan population A	12	Reference Western roan
Western roan population B	14	Reference Western roan
Western roan population C	6	Reference Western roan
Rest of Africa roan population A, Northern Cape	80	Reference rest of Africa roan
Rest of Africa roan population B, North West	18	Reference rest of Africa roan
Known hybrids, Limpopo	8	Known hybrids
Putative hybrid populations, Limpopo	15	Putative hybrids

### Microsatellite markers

We selected nine cross-species microsatellite markers (HN60, HN02, HN17, HN27, HN113, HN58, HN09, HN12 and HN13) that were previously characterised in sable antelope (*Hippotragus niger*) by Vaz Pinto [6] and 12 cross-species microsatellite markers (BM3517, BM203, SPS113, BM1818, OARFCB304, CSSM19, ILST87, BM719, BM757, OARCP26, OARFCB48, INRA006) that were developed for domestic livestock [20–27]. In addition, species-specific microsatellite markers were developed from non-admixed East, Central and Southern African roan using a Next Generation Sequencing approach. The Nextera^®^ DNA Sample Preparation Kit (Illumina, Inc., San Diego, California, USA) was used to create a paired-end library followed by sequencing on the MiSeq^™^ sequencer (Illumina, Inc., San Diego, California, USA) using 2 x 300 bp chemistry (MiSeq^™^ Reagent kit v3 –Illumina Inc., San Diego, California, USA). Library construction and sequencing was carried out at the Agricultural Research Council Biotechnology Platform (Onderstepoort, Gauteng, South Africa). FastQC version 0.11.4 [28] was used to check the quality of the raw sequence reads and Trimmomatic version 0.36 [29] was used to trim and remove the adaptors from the raw sequence reads. Trimmomatic was used with the following settings; HEADCROP:30, CROP:200, SLIDINGWINDOW 15:30, MINLEN:180. Tandem Repeat Finder version 4.09 [30] was used to search the remaining reads for microsatellite motifs and Batchprimer3 software [31] was used to design primer pairs flanking the repeat regions.

### Polymerase Chain Reaction (PCR) and genotyping

DNA extractions were performed using the Qiagen DNeasy^®^ Blood and Tissue Kit (Qiagen GmbH, Hilden, Germany) following the manufacturer’s protocols. Polymerase Chain Reaction (PCR) amplification was conducted in 12.5 μl reaction volumes consisting of AmpliTaq^®^ DNA polymerase (Roche Molecular Systems, Inc) forward and reverse primers (0.5 μM each), and 50 ng genomic DNA template. The conditions for PCR amplification were as follows: 5min at 95°C denaturation, 35 cycles for 30s at 95°C, 30s at 50–62°C (primer-specific annealing temperatures) and 30s at 72°C, followed by extension at 72°C for 10min in a T100^™^ Thermal Cycler (Bio-Rad Laboratories, Inc. Hercules, CA, USA). PCR products were run against a Genescan^™^ 500 LIZ^™^ internal size standard on an ABI 3130 Genetic Analyzer (Applied Biosystems, Inc., Foster City, CA, USA). Samples were genotyped using GeneMapper v. 4.0 software (Applied Biosystems, Inc., Foster City, CA, USA).

### Genetic diversity

Understanding the diversity within groups provide valuable information to identify hybrid individuals. To this end, genetic diversity was evaluated for each group separately (the two different ESUs, known hybrids, and putative hybrids). MICRO-CHECKER [32] was used to detect possible genotyping errors, allele dropout and null alleles. The mean number of alleles per locus (A), allelic richness (AR), observed heterozygosity (Ho), unbiased heterozygosity (H_z_ = expected heterozygosity adjusted for unequal sample sizes) [33] and number of private alleles per reference group (N_P_) was calculated with GenAlEx 6.5 [34,35]. Arlequin 3.5 [36,37] was used to test for deviations from expected Hardy-Weinberg (HW) proportions of genotypes (Markov Chain length of 105 and 100,000 dememorization steps) and to evaluate loci for gametic disequilibrium (with 100 initial conditions followed by ten permutations, based on the exact test described by Guo and Thompson [38]. Associated probability values were corrected for multiple comparisons using Bonferroni adjustment for a significance level of 0.05 [39]. In addition, to determine the discriminatory power of the combined loci, the P_ID_ was calculated using GenAlEx 6.5 [34,35]. Finally, inbreeding (F_IS_) and average pairwise relatedness was calculated for the reference groups, between individuals within populations was calculated using GenAlEx 6.5 [34,35] and the R package Demerelate version 0.9–3 (using 1,000 bootstrap replications) [40]. Demerelate uses the reference populations to create random populations of offspring (full-siblings and half-siblings) and random non-related individuals. The package uses different indices to calculate relatedness (Mxy–genotype sharing, was used for this study) and finally, pairwise t-tests are used to calculate whether the reference populations are significantly different in mean relatedness compared to the full-siblings, half-siblings and non-related individuals created by the package.

### Population structure and admixture analysis

To estimate the degree of genetic differentiation between populations, we performed an analysis of molecular variance (AMOVA) and conducted pairwise F_ST_ comparisons among populations in ARLEQUIN version 3.5 [36,37] using standard AMOVA computations and 1,000 permutations. We used two approaches to assess population structure, namely a Bayesian clustering approach implemented in STRUCTURE version 2.3.4 [41–44] and a Principal Component Analysis (PCA). STRUCTURE was used for the identification of genetic clusters and individual assignment of non-admixed animals as well as putative hybrid individuals and was run using a model that assumes admixture, correlated allele frequencies and without prior population information for five replicates each with K = 1–6, with a run-length of 700,000 Markov Chain Monte Carlo repetitions, following a burn-in period of 200,000 iterations. The five values for the estimated ln(Pr (X|K)) were averaged, from which the posterior probabilities were calculated. The K with the greatest increase in posterior probability (ΔK) [45] was identified as the optimum number of sub-populations using STRUCTURE HARVESTER [46]. The membership coefficient matrices (Q-matrices) of replicate runs for the optimum number of sub-populations was combined using CLUMPP version 1.1.2 [47] with the FullSearch algorithm and G′ pairwise matrix similarity statistics. The results were visualized using DISTRUCT version 1.1 [48]. From the selected K value, we assessed the average proportion of membership (*qi*) of the sampled populations to the inferred clusters. Individuals (parental or admixed classes) were assigned to the inferred clusters using an initial threshold of *qi* > 0.9 [49]. PCA for the complete data set was achieved using the R package Adegent version 2.1.1 [50].

### Maximizing the accuracy of assignments

To determine which threshold *qi*-value (hybridization or admixture index from clustering algorithms used in STRUCTURE) would maximize the accuracy of assignment, simulated genotypes were created using HYBRIDLAB [51]. Genotypes of non-admixed Western roan antelope (n = 30), and animals from East, Central and Southern Africa (n = 30) with *qi* > 0.90 (from STRUCTURE-based analysis) were used as parental (P1) populations to create the simulated hybrid genotypes (see [49]). A dataset consisting of 480 individuals were created consisting of 60 each belonging to non-admixed Western roan antelope, non-admixed Eastern, Central and Southern roan antelope, F1 hybrids, F2 hybrids, backcrosses of F1 with Western roan (BC-Western roan), backcrosses of F1 with East, Central and Southern roan antelope (BC-East, Central and Southern roan), double backcross of BC-Western roan with Western roan (2x BC-Western roan) and double backcross of BC-rest of Africa roan with East, Central and Southern roan antelope (2x BC-East, Central and Southern roan). The simulated dataset was analysed with STRUCTURE version 2.3.4 [41,43,44] using the admixed model, correlated allele frequencies and without prior population information for five replicates each with K = 2, a run-length of 700,000 Markov Chain Monte Carlo repetitions and a burn-in period of 200,000 iterations.

## Results

### Species-specific microsatellite markers

In this study, species specific microsatellite markers were successfully developed using DNA extracted from non-admixed roan antelope (i.e., animals of known provenance). Read lengths of 2 x 301 bp (2 x 3,306,938) were obtained and after trimming, the remaining reads ranged from 180 to 200 bp (2 x 1,596,026). A total of 14 unique loci were identified, of these only six were polymorphic and consistently amplified animals from both ESUs (Table 2).

**Table 2:** List of six species-specific microsatellite loci developed in *Hippotragus equinus*: F = forward primer; R = reverse primer; bp = base pairs. GenBank accession numbers are MN699986-MH699992.

Marker name	Sequence (5’-3’)	Repeat unit	Fluorescent dye label	Product size in bp
RAO2118F	tgccattctgtcctttctca	(TG)_12_	FAM	120
RAO2118R	agggacatgacttatgactgaaca			
RAO4116F	agcaatcctttgcacgaaat	(AC)_12_	VIC	124
RAO4116R	atgccagatttgggtgacat			
RAO7593F	tgcagccagattctttacca	(TG)_14_	NED	120
RAO7593R	caccagaggagcccatatgta			
RAO4422F	cacgagttgttggctgaatg	(AC)_15_	FAM	118
RAO4422R	ctcaggctaacccacaatgc			
RAO13910F	gttgagacctgggcaatgat	(AC)_12_	PET	119
RAO13910R	actaaaggaccgctctgctc			
RAO11139F	cattgagaatcagcgtcctg	(AC)_14_	NED	115
RAO11139R	tttccgtacgcctcagaatc			

### Genetic diversity and relatedness

**Fig 1:**
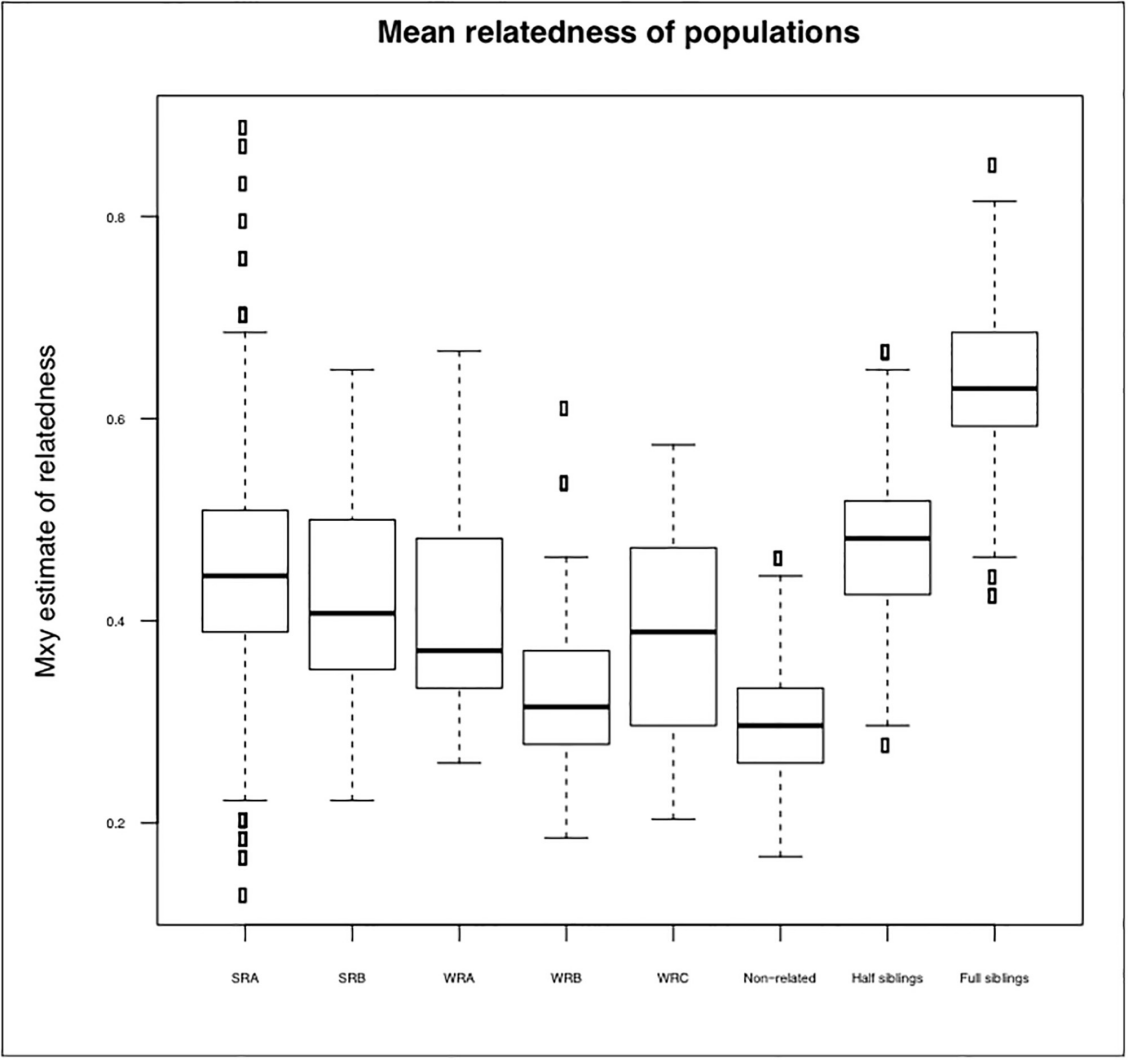
Mean relatedness of rest of Africa roan and Western roan. WRA = Western roan population A, WRB = Western roan population B, WRC = Western roan population C, SRA = Rest of Africa roan population A, rest of Africa population B, HYB = known hybrids, PTH = putative hybrids.

Deviations from HW equilibrium (HWE) were not consistent across populations, with significant deviations from HWE being observed only in the East, Central and Southern African roan populations. In the East, Central and Southern Africa roan population A (Northern Cape Province), 11 loci (BM3517, BM719, OARFCB48, CSSM19, BM1818, BM757, SPS113, INRA006, OARFCB304, RAO4116 and HN27) deviated from HWE. In addition, two loci (BM3517 and SPS113) deviated from HWE in East, Central and Southern Africa roan population B (North West Province) following Bonferroni correction. These markers indicated significant heterozygote deficit in the respective populations with H_o_ values lower than H_z_ values, which may be an indication of the presence of possible null alleles. However, null alleles were only observed in six markers (BM3517, BM719, SPS113, INRA006, RAO4116 and HN27) from the East, Central and Southern African roan group. Significant linkage disequilibrium (LD) was also observed only in the East, Central and Southern African group. These departures from equilibrium may be because of substructure in this group (see [15], which described three mitochondrial DNA groups within this larger ESU), or because of inbreeding. To further investigate the possible causes of heterozygote deficiency, we estimated the overall inbreeding coefficient per population with positive estimates only being observed in the East, Central and Southern African roan group (F = 0.102). In addition, analysis of the overall population relatedness was conducted, as mating among close relatives may cause heterozygote deficiency. As shown in Fig 1, both groups included related individuals, with the overall population relatedness being higher in the East, Central and Southern African roan group (average = 74%) compared to the West African animals (average = 39%). However, relatedness was not at the level of half-siblings and full siblings.

Genetic diversity for each population is summarized in Table 3. Overall, the genetic diversity in the Western roan populations was higher compared to populations from the East, Central and Southern African ESU, notwithstanding smaller sample sizes. The mean number of alleles (A) ranged from 4.15–6.07 and 4.26–5.70, while allelic richness (AR) ranged from 3.17–4.18 and 2.97–3.17 in the reference West African group, and East, Central and Southern African roan groups respectively. Observed heterozygosity (H_o_) in the Western roan group ranged from 0.67–0.72 and unbiased heterozygosity (H_z_) from 0.65–0.71 while H_o_ in the East, Central and Southern African roan varied from 0.57–0.63 and H_z_ from 0.605–0.609. The overall genetic diversity for the known hybrid and putative hybrid populations were intermediate compared to the Western and East, Central and Southern roan populations. The putative hybrid population indicated higher levels of genetic diversity compared to the known hybrid population. The P_ID_ for the 27 loci was 5.5^−20^, thus the estimated probability of any two individuals by chance alone sharing the same mulitlocus genotype was 1.8^19^ for the 27 loci combined.

**Table 3:** Genetic diversity estimates for roan antelope (*Hippotragus equinus*).

Samples	No. of samples	Mean no. of alleles per locus (A)	Allelic Richness (AR)	Unbiased Heterozygosity (H_z_)	Observed Heterozygosity (H_o_)	Inbreeding coefficient (F_IS_)
Western roan population A	12	4.926	3.418	0.652	0.673	-0.086
Western roan population B	14	6.074	4.182	0.714	0.709	-0.053
Western roan population C	6	4.148	3.165	0.667	0.719	-0.187
Rest of Africa population A	80	5.704	2.970	0.605	0.570	0.101
Rest of Africa population B	18	4.259	2.834	0.609	0.634	-0.075
Known hybrids	8	4.963	3.425	0.671	0.598	0.032
Putative hybrids	15	6.296	3.889	0.688	0.692	-0.041

### Genetic differentiation and admixture analysis

**Fig 2:**
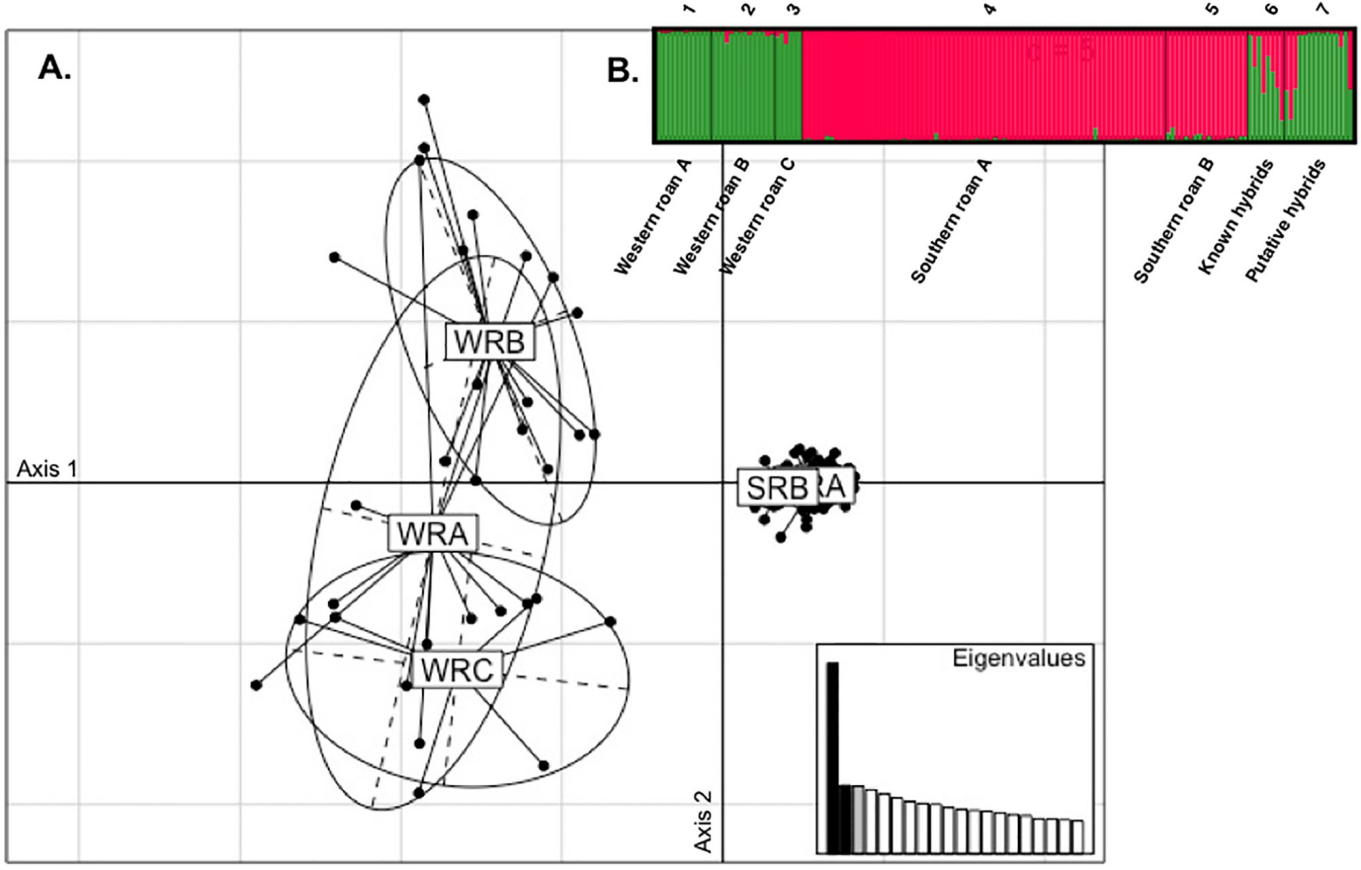
Genetic differentiation analysis between populations based on (A) Principal Component Analysis of Western roan vs East, Central and Southern Africa roan (PCA) and (B) STRUCTURE analysis (performed with K = 2) of Western roan, rest of Africa roan, known hybrids and putative hybrids. WRA = Western roan A, WRB = Western roan B, WRC = Western roan C, SRA = rest of Africa A and SRB = rest of Africa B.

The final dataset included 27 microsatellite loci that yielded a total of 267 alleles, with the number of alleles ranging from 3 to 17 per locus. A total of 27 alleles were unique to the West African roan group, while 27 were found exclusively in the East, Central and Southern African group (Table 4). An analysis of molecular variance (AMOVA) unequivocally retrieved the two distinct groups (corresponding to the two ESUs reported by Alpers [16]; F_ST_ = 0.165, P < 0.001), validating our two reference groups. Principle component analysis similarly revealed a clear separation between the West African versus East, Central and Southern Africa roan (Fig 2A) with 9.3% separation for axis 1 and 3.5% for axis 2. The two distinct genetic clusters (K = 2) were supported by the Bayesian assignment analysis (Fig 2B, S1 Fig). West African versus East, Central and Southern African roan antelope were assigned to two distinct clusters with individual coefficient of membership (*qi*) for non-admixed Western roan *qi* > 0.881 and for non-admixed East, Central and Southern Africa roan *qi* > 0.883.

**Table 4:** Private alleles in loci and allele frequency in Western and rest of Africa roan.

Population	Locus	Allele	Frequency	Population	Locus	Allele	Frequency
Western roan	BM203	230	0.040	Rest of Africa roan	BM203	240	0.005
Western roan	Oarcp26	146	0.031	Rest of Africa roan	BM719	169	0.005
Western roan	Oarcp26	148	0.047	Rest of Africa roan	BM719	177	0.074
Western roan	OARFCB48	176	0.078	Rest of Africa roan	Oarcp26	118	0.010
Western roan	BM1818	280	0.089	Rest of Africa roan	Oarcp26	124	0.015
Western roan	BM757	180	0.031	Rest of Africa roan	BM1818	256	0.058
Western roan	ILST87	153	0.018	Rest of Africa roan	BM1818	278	0.016
Western roan	ILST87	159	0.018	Rest of Africa roan	BM1818	282	0.068
Western roan	RAO4422	115	0.017	Rest of Africa roan	BM1818	288	0.037
Western roan	RAO4422	129	0.017	Rest of Africa roan	INRA006	117	0.077
Western roan	RAO4422	137	0.050	Rest of Africa roan	INRA006	123	0.056
Western roan	RAO4422	141	0.050	Rest of Africa roan	INRA006	125	0.077
Western roan	RAO4422	149	0.017	Rest of Africa roan	INRA006	127	0.337
Western roan	RAO4422	151	0.033	Rest of Africa roan	OARFCB304	115	0.016
Western roan	RAO4422	155	0.033	Rest of Africa roan	OARFCB304	127	0.005
Western roan	RAO4422	159	0.017	Rest of Africa roan	ILST87	121	0.005
Western roan	RAO13910	115	0.031	Rest of Africa roan	ILST87	127	0.005
Western roan	RAO4116	126	0.047	Rest of Africa roan	RAO13910	141	0.040
Western roan	HN02	186	0.063	Rest of Africa roan	RAO11139	102	0.010
Western roan	HN17	202	0.109	Rest of Africa roan	RAO11139	104	0.026
Western roan	HN58	124	0.031	Rest of Africa roan	RAO11139	108	0.072
Western roan	HN58	144	0.016	Rest of Africa roan	RAO4116	112	0.086
Western roan	HN09	152	0.047	Rest of Africa roan	HN09	168	0.005
Western roan	HN09	180	0.031	Rest of Africa roan	HN09	173	0.005
Western roan	HN09	194	0.031	Rest of Africa roan	HN12	185	0.005
Western roan	HN12	171	0.032	Rest of Africa roan	HN12	195	0.005
Western roan	HN12	193	0.032	Rest of Africa roan	HN13	184	0.025

**Fig 3:**
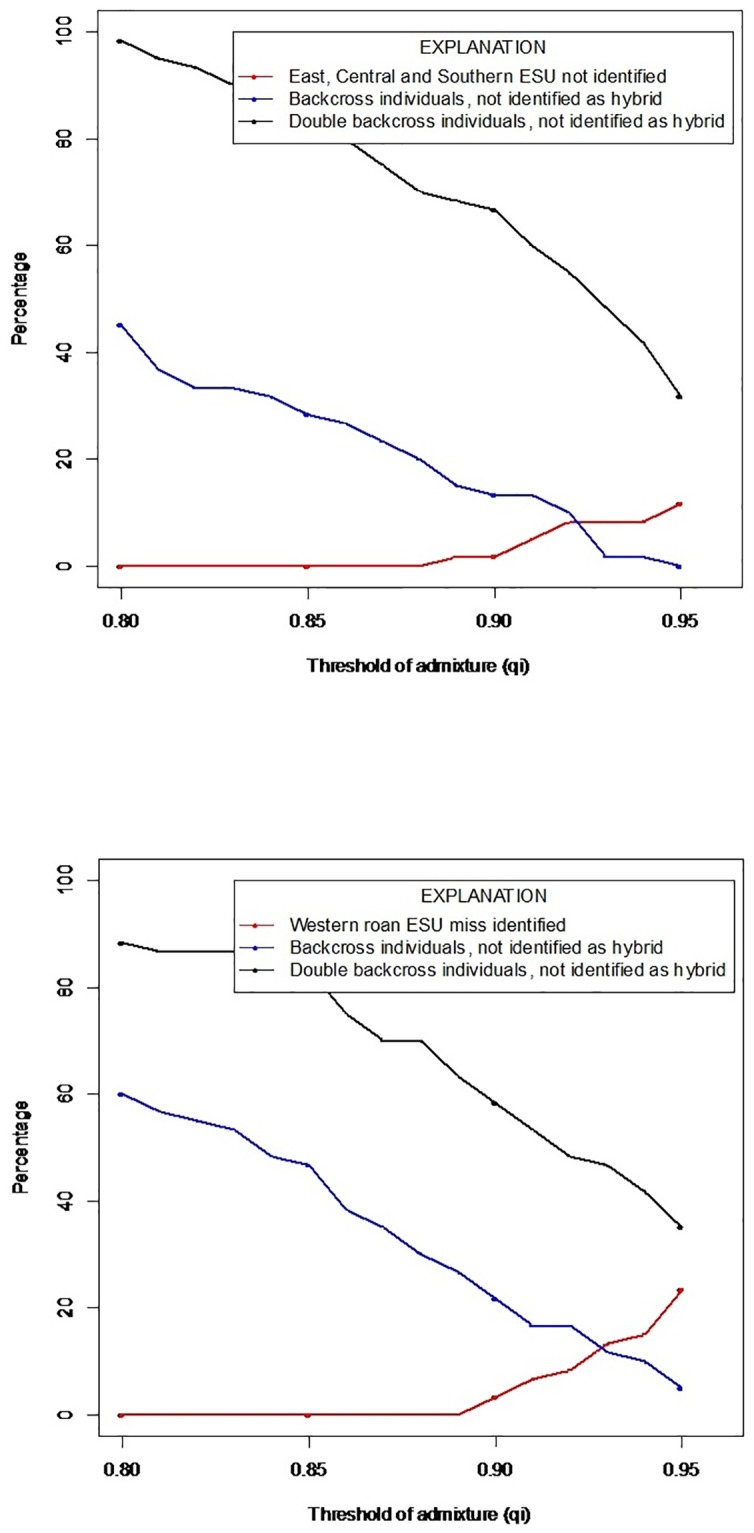
(a) The percentage East, Central and Southern roan individuals (red line), backcross to East, Central and Southern roan individuals (blue line) and double backcross to East, Central and Southern roan individuals (black line) incorrectly identified at different threshold (*qi*) values. (b) The percentage Western roan individuals (red line), backcross to Western roan individuals (blue line) and double backcross to Western roan individuals (black line) incorrectly identified at different threshold (*qi*) values.

On South African farms, game owners often employ selective breeding to achieve specific outcomes. For example, hybrid animals may be backcrossed with pure roan to selectively breed hybrid lineages back to pure. However, the success of detecting hybrid individuals after successive generations of backcrossing can be problematic, thus in this study we additionally assessed whether our markers were able to detect backcrossed animals. Here, we created a simulated dataset to maximize the accuracy of assignment to distinguish between the two non-admixed groups (Western Africa vs East, Central and Southern roan antelope), F1 hybrids, F2 hybrids, F1 BC-Western roan, F1 BC-East, Central and Southern roan, 2x BC-Western roan and 2x BC-East, Central and Southern roan. The threshold of admixture (*qi*) was analysed as a continuous variable and the ability of the marker set to accurately assign individuals was determined at four threshold values (0.8, 0.85, 0.9 and 0.95). STRUCTURE analysis of simulated genotypes generated by HYBRIDLAB indicated that all (100%) of the non-admixed West Africa roan (*qi* = 0.896–0.984) and non-admixed East, Central and Southern African roan (*qi* = 0.887–0.986), F1 hybrids (*qi* = 0.265–0.739) and F2 hybrids (*qi* = 0.182–0.766) genotypes were correctly assigned at selected thresholds of *qi* > 0.80 and *qi* > 0.85 (Fig 3a & 3b). At a selected threshold value of *qi* > 0.90, all F1 and F2 hybrids were correctly assigned while 3.3% of non-admixed Western Africa roan and 1.7% of non-admixed East, Central and Southern African roan were incorrectly identified as hybrid individuals. At a selected threshold value of *qi* > 0.95, all F1 and F2 hybrid individuals were correctly assigned, however, 23.3% of non-admixed Western roan and 11.7% of non-admixed East, Central and Southern African roan were incorrectly identified as hybrid individuals.

Our ability to distinguish non-admixed roan from backcrossed individuals may be problematic in some instances with 60% of BC-Western roan individuals (*qi* = 0.600–0.969) and 45% of BC-East, Central and Southern African roan individuals (*qi* = 0.528–0.946) being incorrectly assigned as non-admixed individuals at *qi* > 0.80. The percentage of incorrectly assigned individuals decreases with an increase in the threshold value for both BC-Western roan (46.7% at *qi* = 0.85; 23.3% at *qi* = 0.90; 5% at *qi* = 0.95) and BC-East, Central and Southern African roan individuals (28.3% at *qi* = 0.85; 13.3% at *qi* = 0.90; 0% at *qi* = 0.95) (Fig 3a & 3b). In addition, when considering the ability of the marker set to detect double backcrossed individuals, at a threshold value of *qi* > 0.8, 88.3% 2x BC-Western roan (*qi* = 0.639–0.978) and 98.3% 2x BC-East, Central and Southern African roan (*qi* = 0.789–0.982) were incorrectly identified as non-admixed individuals. The incorrect identification of double backcross individuals as non-admixed individuals also decreases with an increase in the threshold value for 2x BC-Western roan (83.3% at *qi* = 0.85; 58.3% at *qi* = 0.90; 36.7% at *qi* = 0.95) and 2x BC-East, Central and Southern roan (85% at *qi* = 0.85; 66.7 at *qi* = 0.90; 35 at *qi* = 0.95) (Fig 3a & 3b).

Based on the simulation results, the threshold of *qi* > 0.85 was selected for the analysis of the non-admixed populations, known hybrids and putative hybrids. At this selected threshold, all non-admixed Western, non-admixed East, Central and Southern roan, F1 hybrids and F2 hybrids were correctly identified, whereas some backcross and double backcross individuals were correctly assigned. With an increase in the threshold value a larger percentage of backcross and double backcross individuals would be identified however a higher percentage non-admixed Western roan and East, Central and Southern roan would be incorrectly identified as hybrid individuals. The *qi* > 0.85 threshold was applied to the real dataset and all non-admixed Western roan and all non-admixed East, Central and Southern roan were correctly identified as non-admixed. In addition, six of the eight know hybrids were confirmed as hybrids, with two hybrids being identified as non-admixed Western roan (*qi* = 0.9664 and *qi* = 0.9510 respectively). Analysis of putative hybrids identified four out of 15 animals (27%) as hybrid and 11 (73%) as non-admixed Western roan.

## Discussion

An increasing number of species experience dramatic declining population numbers globally, with ample evidence suggesting that we are entering a mass extinction event. Although the drivers of these population declines are numerous and varied, the underlying root cause inevitably stems from anthropogenic pressures. Not surprisingly, hybridization and admixture of groups with distinct evolutionary trajectories are increasing, raising concerns about the integrity of a large number of species, especially those that experience disproportionately large human interest. For roan antelope, one of Africa's most spectacular large antelope species, this is certainly the case. Although roan antelope numbers are increasing in South Africa (largely because of protection under private ownership), real concerns exist about their genetic integrity given admixture with West African roan antelope, also for export to neighbouring countries. We discuss our results here, and provide some suggestions for roan antelope conservation in South Africa.

### Evidence of hybridization

Using a suite of variable and informative microsatellite markers, we provide evidence of hybridization and introgression between roan antelope naturally occurring in South Africa (East, Central and Southern African origin), and those of West African decent (a separate evolutionary significant unit; see [15]). More problematic, the identification of first and second generation backcrosses with *q*-values close to threshold values strongly suggest that hybrid individuals are viable and fertile; as also suggested from anecdotal evidence from some game farms. Although genetic diversity estimates were moderately higher in the known and putative hybrid individuals, it has previously been reported that F2 hybrids can display reduced fitness as a result of disruption of sets of co-adapted gene complexes by recombination [52,53], thereby weakening the entire gene pool of naturally occurring individuals. Our marker set was able to accurately identify F1 and F2 hybrids as well as non-admixed individuals at thresholds of *qi* > 0.80 and *qi* > 0.85. However, the classification of further backcrosses was less accurate at these thresholds with backcrossed and double backcross individuals being incorrectly classified as non-admixed. The use of higher thresholds (*qi* > 0.90 and *qi* >0.95) did increase the number of individuals correctly identified as backcross and double backcross individuals, however, this also resulted in an increase in the number of non-admixed individuals being incorrectly classified as hybrids. Based on the simulated results a threshold value of qi > 0.85 was thus selected as all non-admixed Western and non-admixed East, Central and Southern roan, F1 and F2 hybrids would be correctly identified and some backcross and double backcross individuals would be correctly identified. In certain instances, backcrossed and double backcrossed individuals extend beyond the detection power of the current microsatellite marker panel.

The minimum number of markers required to accurately and consistently identify backcrosses is currently being debated. Simulation analysis in the grey wolf (*Canis lupus*) that hybridizes with domestic dogs (*C*. *lupus familiaris*) indicated that simply increasing the number of microsatellite markers used does not equate to an increase in the number of correctly identified admixed individuals [54]. It may be important to evaluate single nucleotide polymorphisms (SNPs) with high discriminating power to increase the ability to detect backcrossed and double backcrossed individuals, but in all likelihood thousands of SNPs may be required. Notwithstanding, the marker set described here represents the first step in assessing hybridization in roan antelope, and in the identification of hybrid individuals.

### Conservation management

As signatories to the Convention on Biological Diversity, South Africa has an obligation to conserve the genetic integrity of its biological diversity. Furthermore, admixture between distinct wildlife subspecies is prohibited under national and provincial legislation. Within South Africa, wildlife can be privately owned. There has been some debate about the legal rights of an owner to act in a certain manner with its property, and whether farming with wildlife should be managed and regulated any differently than, for example, agricultural stock such as cattle. Notwithstanding, current international, national and provincial legislation is clear in prohibiting admixture, irrespective of ownership.

The private ownership of biological diversity has been advantageous for a large number of species, and the high commercial value attached to many of these species has undoubtedly aided in their conservation and protection; to the point where a number of species are doing better under private ownership compared with in protected areas or national parks [55]. Roan antelope is a prime example, but others include sable antelope, white and black rhinoceros, and bontebok to name but a few. Unfortunately, many of these species are intensively managed, with selection for specific desired traits. These management practises have unintended consequences, notably a loss of genetic diversity. In our study, a number of loci showed deviations from HWE and linkage disequilibrium; all which can be ascribed to small numbers of founding individuals and genetic drift on farms [56] which may, in the long term, compromise local adaptation [57]. To fully understand the impact that farming practises, notably intensive management and selection, have on wildlife populations, comparisons need to be done with naturally occurring populations on nature reserves.

Currently, the full extent of hybridization in South Africa between roan antelope belonging to the two distinct ESUs is unknown. Laboratory screening for permitting purposes (to either sell, or translocate animals) suggest that the occurrence of widespread introgression is low, and largely confined to specific game farms.

Animals of West African decent are no longer maladapted to South African conditions and have, over the span of 20 years, adapted to local conditions. The question that needs consideration is whether South Africa should safeguard the genetic integrity and genetic variability of both roan ESUs. If historic occurrence is considered, then all West African animals should be removed from South African populations. However, the South African situation has spawned several *ex situ* breeding programmes and agreements and/or animals that could be allowed to be backcrossed to obtain some form of purity, over four or five generations. This might improve genetic variation within the national population, but may not be desirable given that the impact of hybridization on the South African roan full genome is not known. Thus, we recommend the implementation and continuation of strict genetic monitoring for hybridization in roan antelope in South Africa, at least for the foreseeable future. With the microsatellite marker set described here, and using a threshold of *qi* = 0.85, it is possible to detect F1 and F2 hybrids prior to translocation, thereby reducing and ultimately eliminating Western roan antelope alleles in the indigenous roan gene pools. In addition, management of roan in South Africa would benefit from a national meta-population conservation plan to inform translocations and reintroductions and to effectively monitor genetic diversity and further hybridization events.

## Supporting information

S1 Fig a) Probability (-LnPr) of K = 1–6 averaged over 5 runs. b) Delta K values for real population structure K = 1–6.
(PDF)
