# Supplementary figures and images for: Assessing introgressive hybridization in roan antelope (Hippotragus equinus): Lessons from South Africa
Source: PLoS One. 2019 Oct 18;14(10):e0213961. doi: 10.1371/journal.pone.0213961 (PMC6799913; doi:10.1371/journal.pone.0213961)

S1 Fig. a) Probability (-LnPr) of K = 1 – 6 averaged over 5 runs. b) Delta K values for real population structure K = 1 – 6

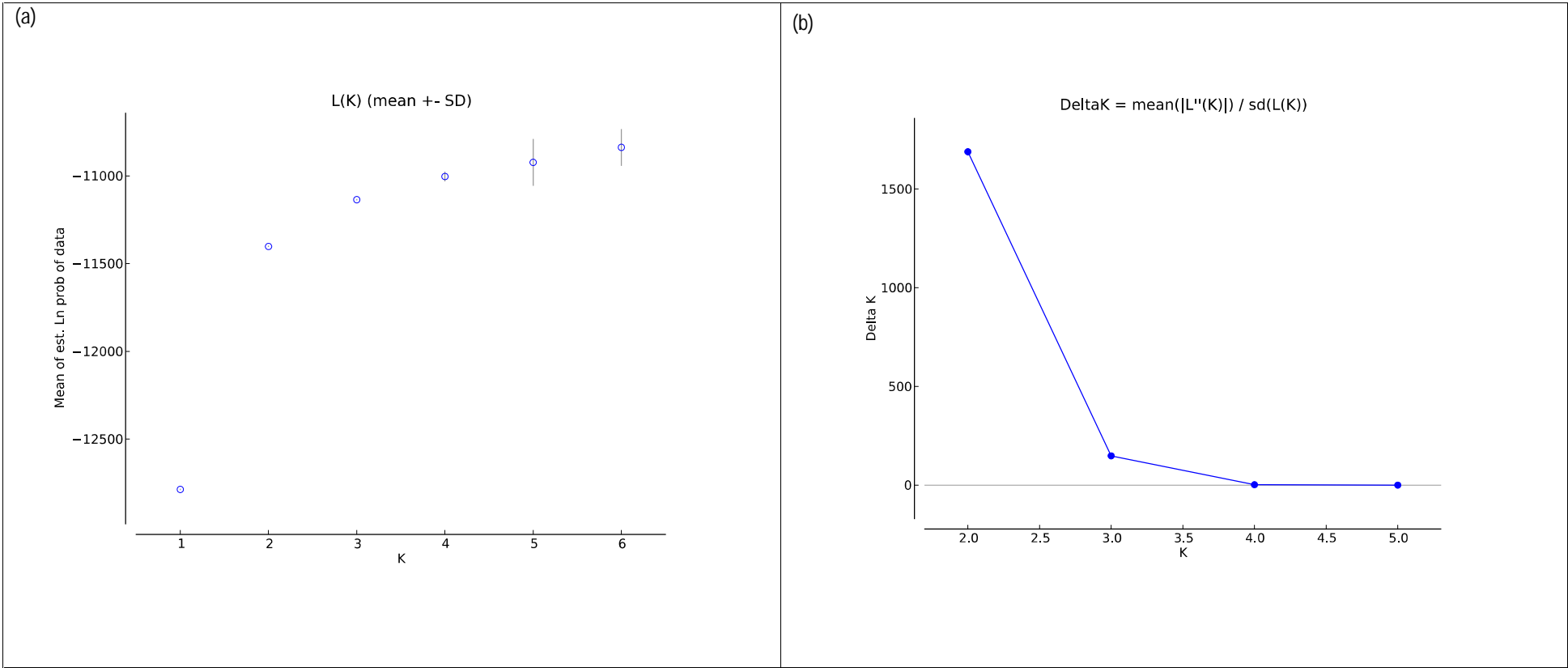

Supplement: S1 Fig — a) Probability (-LnPr) of K = 1–6 averaged over 5 runs. b) Delta K values for real population structure K = 1–6. (PDF) [file pone.0213961.s001.pdf]
